# Engineering Potato Starch with a Higher Phosphate Content

**DOI:** 10.1371/journal.pone.0169610

**Published:** 2017-01-05

**Authors:** Xuan Xu, Xing-Feng Huang, Richard G. F. Visser, Luisa M. Trindade

**Affiliations:** 1 Wageningen UR Plant Breeding, Wageningen University and Research, Wageningen, The Netherlands; 2 National Centre for Vegetable Improvement (Central China), Key Laboratory of Horticultural Plant Biology, Ministry of Education, Huazhong Agricultural University, Wuhan, China; Shanghai Institutes for Biological Sciences, CHINA

## Abstract

Phosphate esters are responsible for valuable and unique functionalities of starch for industrial applications. Also in the cell phosphate esters play a role in starch metabolism, which so far has not been well characterized in storage starch. Laforin, a human enzyme composed of a carbohydrate-binding module and a dual-specificity phosphatase domain, is involved in the dephosphorylation of glycogen. To modify phosphate content and better understand starch (de)phosphorylation in storage starch, laforin was engineered and introduced into potato (cultivar Kardal). Interestingly, expression of an (engineered) laforin in potato resulted in significantly higher phosphate content of starch, and this result was confirmed in amylose-free potato genetic background (*amf*). Modified starches exhibited altered granule morphology and size compared to the control. About 20–30% of the transgenic lines of each series showed red-staining granules upon incubation with iodine, and contained higher phosphate content than the blue-stained starch granules. Moreover, low amylose content and altered gelatinization properties were observed in these red-stained starches. Principle component and correlation analysis disclosed a complex correlation between starch composition and starch physico-chemical properties. Ultimately, the expression level of endogenous genes involved in starch metabolism was analysed, revealing a compensatory response to the decrease of phosphate content in potato starch. This study provides a new perspective for engineering starch phosphate content *in planta* by making use of the compensatory mechanism in the plant itself.

## Introduction

Starch is the predominant storage carbohydrate in higher plants and is a semi-crystalline composite substrate consisting of two biopolymers, amylose and amylopectin. Amylose is an amorphous, essentially linear glucan polymer with α-1,4 linked glucose residues, while amylopectin is a highly branched molecule built of α-1,4 linked glucose residues as backbone and 5% α-1,6 branches [1]. In addition to amylose and amylopectin, native starches contain small amounts of phosphate groups monoesterified to the glucose residues [2–5]. Most of the phosphate groups are bound to the amylopectin fraction at the C-6 (~70%) and C-3 (~30%) positions of the glucose units [6, 7]. C-6 phosphoesters are added by the glucan water dikinase (GWD1), whereas C-3 phosphoesters are catalysed by the phosphoglucan water dikinase (GWD3/PWD) [8–11]. Studies in *Arabidopsis* have indicated that phosphoglucan phosphatase starch excess 4 (SEX4) cooperates with like-SEX4 1 and 2 (LSF1 and LSF2) proteins to remove phosphate groups [12–14]. This reversible phosphorylation is essential for starch degradation in leaves. In contrast, it is not known whether the dephosphorylation occurs in storage starches, and in case it does the mechanism of phosphate removal is unknown.

Starch phosphate content differs considerably between botanical origins [2, 15]. For instance, cereal endosperm starch has less than 0.01% covalently linked phosphate, while potato tuber starch contains significantly higher phosphate content with approximately 0.5% glucose residues being phosphorylated [2, 5]. It has been shown that phosphate content affects physico-chemical properties and the end-uses of starches, such as starch pasting properties, gel strength and clarity, stickiness and viscosity [3]. Hence, modification of starch phosphate content is a prerequisite for some industrial applications, such as internal sizing in paper making, special flocculation effects in mining, and water treatment [16]. In industry, a common method to increase starch phosphate content is chemical phosphorylation, but this process requires high amounts of energy and produces pollutant waste.

To preclude the disadvantages of post-harvest modifications of starch, many studies have focused on ways of producing starches with different phosphate content directly *in planta* [15, 17]. One of the alternatives is to manipulate the endogenous genes involved in starch biosynthesis through genetic engineering. Up to date, most known endogenous genes, including granule-bound starch synthase (*GBSSI*), glucan water dikinase (*GWD1*), phosphoglucan water dikinase (*GWD3*), starch-branching enzyme (*SBE*), soluble starch synthases (*SSS*), have been overexpressed or down-regulated (antisense or co-suppression approaches) in plants to obtain starches with altered phosphate content [18–20]. For instance, *GWD1* has been overexpressed in rice [21], maize [22], wheat [23] and barley [24], resulting in increased starch phosphate content. In potato, silencing of *GWD1* has resulted in the reduction of both phosphate content in starch and cold-sweetening in tubers [25], and in wheat, the inhibition of *GWD1* has led to a lower phosphate content and an increased seed yield and plant biomass [26].

Another strategy is to introduce heterologous enzymes that are able to modify starch composition and structure and in this way produce starches with novel properties [15]. Phosphate content can be removed by phosphatases, which are widely spread throughout different kingdoms. One of such enzymes is Laforin, a dual specificity phosphatase required for normal glycogen metabolism in vertebrates. Mutations in the laforin gene lead to Lafora disease, an autosomal recessive neurodegenerative disorder causing severe epilepsy and death [27–29]. Lafora disease is characterized by the accumulation of Lafora bodies (LBs), insoluble deposits containing high phosphate content that closely resemble amylopectin [30]. Interestingly, laforin has structural similarities to SEX4, containing a dual-specificity phosphatase (DSP) and an N-terminal carbohydrate-binding module (CBM) of the CBM20 subtype. Moreover, it is a functional equivalent of SEX4 [31], and is unique in its ability to remove phosphate from amylopectin in vitro [32]. Together, these findings suggest that laforin has a great potential to modulate phosphate content in potato starch, and could help to explore the mechanism of phosphoglucan metabolism in potato starch.

In this study, the human laforin gene, and modifications of it, were introduced into potato plants to modify phosphate content of starch. The effects of the (engineered) laforin on phosphate content, composition and properties of potato starch are presented and discussed.

## Materials and Methods

### Construct preparation

Three constructs were made in this study, i) pBIN19/DSP for expression of the dual-specificity phosphatase (DSP) domain of laforin alone, ii) pBIN19/CBM20-DSP for the full-length laforin gene expression, iii) pBIN19/SBD-DSP for the expression of the DSP domain fused to a starch-binding domain (SBD) that is derived from *Bacillus circulans* cyclodextrin glycosyltransferase (CGTase) [33–35]. The potato granule bound starch synthase (GBSSІ) promoter and the GBSSI transit peptide were used for tuber-specific expression and amyloplast entry of proteins, respectively. All constructs were validated by sequence analysis.

**Fig 1:**
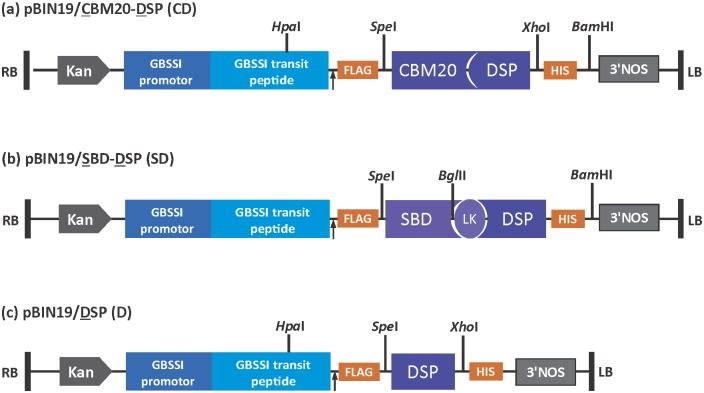
Schematic depiction of binary vector constructs used in this study. Genes were cloned in frame with GBSSI transit peptide to allow amyloplast targeting and were driven by GBSSI promoter for tuber-specific expression. CBM20 and DSP represent the carbohydrate-binding module 20 domain and a dual specificity phosphatase of laforin protein. RB and LB stand for right and left borders, respectively. SBD, LK, Kan and 3’NOS stand for starch binding domain of cyclodextrin glycosyltransferase from *Bacillus circulans*, linker, kanamycin resistant gene and NOS terminator, respectively. The arrow represents cleavage site of the transit peptide. FLAG and HIS are two tags for protein quantification and *Hpa*I, *Spe*I, *Xho*I, *Bgl*II and *Bam*HI are restriction enzyme sites.

For the assembly of the pBIN19/CBM20-DSP construct, the laforin-encoding fragment was obtained by PCR amplification with the primers 5’- TA**CCATG****G**ACTACAAAGACGATGACGATAAAACTAGTATGCGCTTCCGCTTTGGGGTG-3’ (FLAG-encoding sequence underlined) and 5’-GA**GGATCC**ACGGATCTCAGTGGTGGTGGTGGTGGTGCTCGAGCAGGCTACACACAGAA-3’ (HIS-encoding sequence underlined), which contained *Nco*I and *Bam*HI sites at 5’ ends (in bold), respectively. This amplified laforin fragment, containing an N-terminal FLAG tag and a C-terminal HIS tag, was cloned into *Nco*I/*Bam*HI restriction sites of pUC19/SBD2 (S1 Fig), generating the pUC19/CBM20-DSP plasmid. After digestion of this plasmid with *Hpa*I and *Bam*HI, the fragment was inserted into the corresponding sites of pBIN19/SBD2 [36] to generate the pBIN19/CBM20-DSP plasmid (Fig 1).

The linker-DSP fragment was amplified from pBIN19/CBM20-DSP by PCR, with a forward primer containing a *Bgl*II site (5’- AT**AGATCT**TTGGTGGATGGTGTGTA -3’) and a reverse primer containing a *Bam*HI site (5’- GC**GGATCC**AGCAGCCGGATCTCAGTGGTG -3’). The amplified fragment contained a linker, DSP sequence and HIS tag in which the linker was 117 bp fragment of the 3’-end of CBM20 sequence. The amplified fragment was cloned into *Bgl*II/*Bam*HI restriction site of pUC19/SBD2, generating pUC19/SBD-DSP. Furthermore, the fragment was amplified from pUC19/SBD-DSP with a forward primer containing a *Spe*I restriction site (5’-AA**ACTAGT**ATGGCCGGAGATCAGGTCAG-3’) and a reverse primer containing a *Bam*HI site (5’- GC**GGATCC**AGCAGCCGGATCTCAGTGGTG -3’). After digestion of this fragment with *Spe*I and *Bam*HI, the fragment was inserted into the corresponding sites of pBIN19/CBM20-DSP to generate the pBIN19/SBD-DSP plasmid (Fig 1).

The pBIN19/DSP construct was made based on pBIN19/CBM20-DSP plasmid. The DSP-encoding fragment was amplified by PCR with a forward primer, containing a *Spe*I restriction site (5’-AA**ACTAGT**CATTATTCAAGAATTCTACCAAATATCT-3’) and a reverse primer, containing an *Xho*I site (5’- TC**CTCGAG**CAGGCTACACACAGAAGAA -3’). The amplified fragment was inserted into corresponding sites (*Spe*I and *Xho*I) of pBIN19/ CBM20-DSP, resulting in pBIN19/DSP plasmid (Fig 1).

### Transformation and regeneration

All three binary vectors, pBIN19/CBM20-DSP, pBIN19/SBD-DSP and pBIN19/DSP (Fig 1), were transformed into an amylose-containing potato (cv. Kardal, tetraploid) and amylose free mutant (*amf*, diploid) according to Visser, Stolte and Jacobsen [37]. About thirty independent plantlets of each construct as well as control plants (untransformed plants and transformed plants with empty vector) were multiplied to five plants by culturing nodal explants according to the procedure described in Visser, Stolte [37]. All plants were grown under standard greenhouse conditions (16 h light at 20°C and 8 h dark at 18°C) till maturity and tubers were harvested at 18 weeks. No differences were detected between untransformed lines and transformed plantlets with empty vector, and therefore they will be further referred to as control or UT. Transformed potato plants from Kardal background were labelled CDxx, SDxx and Dxx in which CD, SD and D stand for full-length laforin protein, DSP with an SBD, and DSP alone, respectively, xx represents the number of transgenic line. Transformed potato plants from *amf* background were labelled *amf*CDxx, *amf*SDxx and *amf*Dxx.

### Starch isolation

To minimize individual variation, tubers used for starch isolation were obtained by pooling all tubers of five plants harvested from the same transgenic clone. Starches were isolated according to the procedure described in Huang, Nazarian-Firouzabadi [33].

### Western dot blot and protein analysis

The amount of granule-bound fusion protein of each transformant was investigated by western dot blot analysis. About 50 mg of starch were heated at 100°C for 5 min with 400 μl of a 2 × SDS sample buffer containing 5% β-mercaptoethanol [38]. After cooling to room temperature, the supernatant was transferred to a 96-well format dot-blot manifold (Schleicher & Schuell, Keene, NH). The dot-blot manifold was connected to a water pump, and a vacuum was applied for 5 min until all samples were impacted on the nitrocellulose membrane (Bio-rad).

The dot blots were blocked overnight at 4°C in Tris-buffered saline (TBS is 20 mM Tris, 500 mM Nacl pH 7.5) with 0.1% Tween-20 (TBST) containing 5% (w/v) dry powdered non-fat milk. Membranes were then incubated for 2 h at room temperature with a 1:1000 dilution of the anti-SBD antibody [35] in 3% non-fat milk in TBST, followed by 5 rinses in TBST. The membrane was then incubated with a 1:5000 dilution of horseradish-peroxidase-conjugated anti-(rabbit IgG) (Cat # A0545, Sigma) in TBST buffer with 3% (w/v) of non-fat milk for 1 h at room temperature. After 5 times rinses in TBST, the protein was detected with a West Femto supersignal (Cat # 34094, Thermo Scientific) [35].

To examine GBSSI abundance, proteins were stained with Coomassie brilliant blue R-250 after separation by SDS polyacrylamide gel electrophoresis (SDS-PAGE).

### Total phosphate content

Total phosphate content in starch was determined essentially according to the method of Morrison [39] with some modifications. About 20 mg of dry starch were suspended in 250 μl of 70% (w/w) HClO_4_ and completely charred at 250°C for 25 min. The solution was clarified by adding 50 μl 30% (w/v) H_2_O_2_ and gently boiled for 2 min. Once the solution had cooled, water was added to a final volume of 2 ml and 100 μl of the sample was transferred into a 96-well microtiter plate, followed by adding 200 μl of colour reagent [0.75% (w/v) (NH_4_)_6_M_O7_O_24_.4H_2_O, 3% (w/v) FeSO_4_.7H_2_O and 0.75% (w/v) SDS dissolved in 0.375 M H_2_SO_4_]. Absorbance at 750 nm was then measured in a Model 680 XR Microplate Reader (Bio-Rad, US), and a calibration curve was used to calculate the concentration in nmol PO_4_/mg starch.

### Analysis of the morphology and physico-chemical properties of starch granules

All analyses conducted in this manuscript have been performed in duplicate unless indicated otherwise.

Starch samples were stained with 20×diluted Lugol’s solution (1% I_2_/KI) and were then investigated with light microscopy (LM, Axiophot, Germany). Further, scanning electron microscopy (SEM) Phenom^™^ (FEI, The Netherlands) was used to examine the detailed morphology on the granule surface. Starch samples of approximately 1 mg were evenly distributed on a carbon tabs (Agar Scientific, UK) and mounted onto 12.70 mm aluminium specimen stubs (Agar Scientific, UK), followed by coating with gold using a sputter coater (EMITECH K550X; Quorum Technologies, UK). All images were digitally recorded.

Particle size distribution and gelatinization properties of starches were analysed as described in Ji, Vincken (35). The onset (T_o_), peak (T_p_) temperature of gelatinization and the melting enthalpy (Δ*H*, J/g) were determined using Differential Scanning Calorimetry (DSC).

The apparent amylose content was performed according to the procedure described in Hovenkamp-Hermelink, De Vries, Adamse, Jacobsen, Witholt and Feenstra [40].

High-performance anion-exchange chromatography (HPAEC) was used to determine amylopectin chain length distribution. Degree of polymerisation (DP) 6–35 was separated according to the method of Huang, Nazarian-Firouzabadi [33].

Starch moisture content was measured in duplicate using standard methods (AACC Approved Method 44–15.02), which involved oven drying at 105°C for 16 hours.

Starch content was determined as described by Kok-Jacon, Vincken, Suurs and Visser [41].

### Quantitative RT-PCR

Expression levels of (engineered) laforin from all transformants were determined in triplicate by qRT-PCR using the primers listed in S1 Table. Total RNA was extracted from potato tuber samples according to Kuipers, Jacobsen [4] and reverse transcribed using the iScript cDNA synthesis kit from BioRad. qRT-PCR analysis was performed using CFX96 Real-Time PCR machine (BioRad). The total volume of each reaction was 10 μl, containing 50 ng cDNA, 3 μM of each gene-specific primer, and 5 μl SYBR Green Supermix Reagent (BioRad). All reactions were carried out using the following thermal cycling conditions: 3 min of denaturation at 95°C, followed by 45 cycles (15 s at 95°C, 60 s at 60°C). Using the comparative Ct method [42], target genes were expressed relative to *EF1α* [43]. After normalization, data were multiplied by a factor of 10^6^ and then converted to log 10. Ultimately, the resulting value (v) was used to assign transformants to different categories: none (N, v = 0), low (L, 0 < v < 3), medium (M, 3 ≤ v < 4) and high (H, v ≥ 4) expressers.

Five randomly-selected transgenic lines from D, CD and SD series were used for gene expression analysis of key genes involved in the starch metabolism. In total, four starch-degrading genes and seven starch-synthesizing genes were selected according to previous studies, including phosphoglucan phosphatase starch excess 4 gene *SEX4* [44], like-SEX4 gene *LSF1* and *LSF2* [12, 45], starch phosphorylase gene *SP* [46], α-amylase gene *AMY23* and β-amylase genes *BAM1* and *BAM9* [47, 48], isoamylase genes *ISA1*, *ISA2* and *ISA3* [47, 49], starch-branching genes *SBEI* and *SBEII* [50, 51], soluble starch synthase genes *SSSII* and *SSSIII* [18, 52]. The gene information and specific primers used in present study are listed in S1 Table. The relative expression levels were calculated using the *Ef1α* gene as a reference gene, employing the 2^-ΔΔCT^ method.

### Statistical analysis

Significant differences between modified starches and control samples in phosphate content and granule size were assessed by one-way analysis of variance (ANOVA). The least significant difference values were calculated at 5% probability. Statistical significances between gene expression level of transformants and that of the control were determined by using t-test. Inter-relationships between starch components and starch properties were analysed by means of Pearson correlations. All statistical analyses were performed using GenStat (16th edition). Principle component (PCA) analysis was performed using PAST software Package [53].

## Results

### Transgenic plants do not show visible changes in plant architecture or tuber morphology

In total, three transgenic series containing, respectively, DSP (D), CBM20-DSP (CD) and SBD-DSP (SD) were generated by introducing three constructs into potato plants. About thirty independent plants from each transgenic series and the control were multiplied and grown in the greenhouse. Plants were monitored in all stages and no significant differences were observed in plant architecture, tuber morphology nor tuber yield relative to the control plants (data not shown).

### (Engineered) laforin genes are expressed in transformants

**Fig 2:**
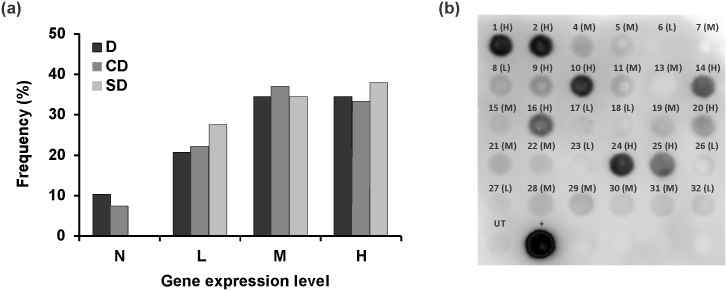
Characterization of transgenic plants. (a) Distribution of the individual transformants over the classes of the (engineered) laforin expression. The qRT-PCR analysis was performed in triplicate on all transformants, which are 27, 26 and 30 lines for D, CD and SD series, respectively. D, CD and SD represent DSP, CBM20-DSP and SBD-DSP transformants, respectively. N, L, M and H stand for none, low, medium and high expressors. (b) Accumulation level of SBD-DSP (SD) in transformants. Protein levels were determined using Western dot blot analysis with an anti-SBD antibody. The number above each dot stands for the different lines, while UT and ‘+’ represent negative and positive control, respectively. The intensity of dots shows the various protein levels. The corresponding gene expression level of each line obtained from qRT-PCR is indicated between brackets. For 90% of lines a good correlation between gene expression level and protein accumulation level was found.

Expression of SD in transformants was examined by qRT-PCR and Western dot blot analysis. According to gene expression level, transformants were classified into four different categories: none (N), low (L), medium (M) and high (H) expressors. All SD lines showed gene expression, and 28%, 34% and 38% of transformants were categorized as L, M and H expressors, respectively (Fig 2a). Furthermore, the result obtained from Western dot blot using a SBD-antibody revealed that the accumulation of the protein correlated well with the mRNA level of the transformants (Fig 2b). For this reason, and because no specific antibody was available for quantification of the DSP domain, the transformants from other two series, D and CD, were only subjected to qRT-PCR analysis to determine the expression of the (engineered) laforin gene. As shown in Fig 2a, similar to SD series, M and H expressers were predominant in these two series. For D series, 10%, 21%, 34% and 34% of the transformants classified as N, L, M and H expressors, respectively, whereas the corresponding values for the CD series were 7%, 22%, 44% and 26%. N-expressors were excluded from further analyses.

### Starch granule morphology and amylose content are altered by the introduction of an (engineered) laforin protein

**Fig 3:**
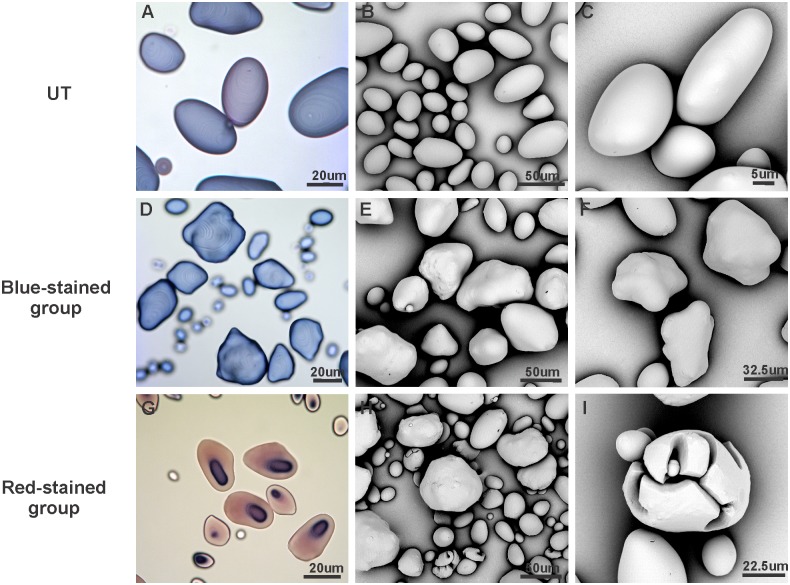
Light micrographs and SEM analyses showing starch granules morphology of control UT (A-C) and modified starches (D-I). Starch granules were stained with a 20× diluted Lugol solution for light microscopy (A, D and G). Two different morphologies were observed in the modified starches from each series. Based on the colour of stained granules, the starches were classified into two categories: blue-stained group (D-F) and red-stained group (G-I).

Light microscopy (LM) and scanning electron microscopy (SEM) were used to investigate how the accumulation of the (engineered) laforin protein affects the starch granule morphology. About 22% of the D transformants, 19% of the CD transformants and 27% of the SD transformants showed red granules with blue cores of varying size when stained with iodine (Fig 3G). Further analyses indicated that both irregular bumpy granules and cracked granules coexisted in each of these modified starches (Fig 3H and 3I), while the starches from other transgenic lines exhibited blue-stained granules with irregular bumpy surface (Fig 3D and 3F) in contrast to the smooth surface observed in the control (Fig 3A–3C). Based on the granule colour, transformants from each series were divided into two groups (red-stained group and blue-stained group) for further analyses. Starch with red-stained granules occurred in transformants with different expression level of (engineered) laforin (data not shown).

In addition, the exhibition of red-stained granules strongly suggests the reduction of amylose content in these starch granules, therefore, the amylose/amylopectin ratio was determined in all modified starches and the apparent amylose content (AM%) was calculated (Table 1). The result revealed that the red-stained starches contained much lower amylose content relative to the control, whereas all blue-stained starches did not show changes in amylose content compared to the control. The amylose level of most red-stained starches was between 2% and 5%, which is comparable to that of the amylose-free potato starch [54]. Moreover, some lines that consisted of a mixture of red and blue-stained granules had intermediate amounts of amylose content (~10%), but still dramatically lower than that of the control (~18%).

**Table 1:** Summary of different starch characteristics determined for the representative modified starches and the control UT.

Line	Class	Colour	P (nmol/mg)	AM (%)	C_starch_ (mg/g FW)	d50 (μm)	T_o_ (°C)	T_p_ (°C)	T_c_ (°C)	Δ*H* (J/g)
**UT**	n.d.	blue	26.7 ± 0.6	18.3 ± 0.2	143.4 ± 4.2	17.5 ± 0.4	66.6 ± 0.2	70.6 ± 0.2	80.3 ± 0.4	20.7 ± 0.3
**D05**	H	red	34.8 ± 0.0	3.3 ± 0.1	131.3 ± 1.9	14.8 ± 1.2	71.0 ± 0.2	76.0 ± 0.1	84.0 ± 0.3	17.2 ± 0.6
**D13**	L	blue	31.8 ± 0.4	17.5 ± 0.4	151.5 ± 1.2	20.6 ± 0.4	68.1 ± 0.1	72.0 ± 0.0	82.2 ± 0.6	18.9 ± 0.7
**CD24**	L	red	33.0 ± 0.3	4.4 ± 0.1	146.7± 2.5	24.2 ± 0.2	69.1 ± 0.1	73.3 ± 0.1	80.9 ± 0.1	17.4 ± 0.2
**CD28**	M	blue	30.8 ± 0.0	18.0 ± 0.5	140.4 ± 2.3	22.5 ± 0.3	66.7 ± 0.2	70.7 ± 0.2	79.4 ± 0.0	18.2 ± 0.2
**SD07**	M	red	35.5 ± 0.0	3.1 ± 0.2	149.5 ± 1.0	24.5 ± 0.4	69.4 ± 0.1	73.5 ± 0.1	81.2 ± 0.2	17.5 ± 0.7
**SD16**	H	blue	30.1 ± 0.1	16.7 ± 0.7	140.1 ± 0.0	18.9 ± 0.1	66.1 ± 0.3	70.0 ± 0.3	79.1 ± 0.0	19.6 ± 0.5

Two representative starches of each series with blue or red-stained granules are presented. Gene-expression class (Class), starch granule colour (Colour), total phosphate content (P), starch apparent amylose content (AM), starch content (C_starch_), median granule size (d50), starch gelatinization temperature (T_o_, T_p_ and T_c_) and gelatinization enthalpy (Δ*H*) are shown. Data (mean ± S.D.) are the average of two or three independent measurements. n.d., not detected.

**Fig 4:**
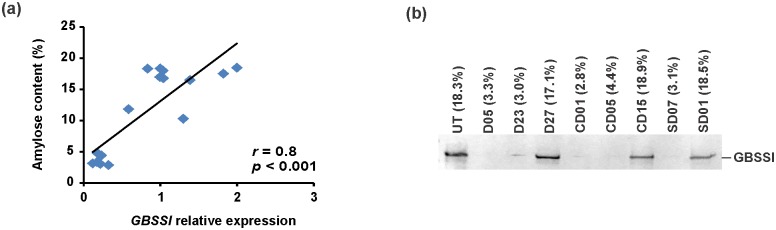
The suppression of GBSSI in transformants with red-stained granules. (a) The correlation between amylose content and the relative expression of granule-bound starch synthase I (*GBSSI*). The qRT-PCR analysis was performed on control UT and 5 random-selected transgenic tubers from each series, containing transformants with 8 red-stained starches and 7 blue-stained starches. (b) Western blot analysis of GBSSI abundance in starches from different transformants. The corresponding amylose content of each line is shown between brackets.

These findings raised a question regarding the expression of *GBSSI* in transformants, as *GBSSI* is responsible for the amylose synthesis. Hence, *GBSSI* expression was investigated for transformants from each series, containing transformants with eight red-stained starches and seven blue-stained starches in total. The results revealed that amylose content strongly correlates with relative expression of *GBSSI* (*r* = 0.8, *p* < 0.001, Fig 4a). Furthermore, eight lines with different expression levels of *GBSSI* were selected to perform SDS-PAGE, followed by staining with Coomassie blue. The correlation between amylose content and GBSSI abundance was observed (Fig 4a and 4b). These results showed that the decrease in amylose content in red-stained starches is a consequence of GBSSI suppression in transformants.

### Laforin expression results in higher phosphate content

The total phosphate content in starch granules of each transgenic line was determined. Interestingly, nearly all the starches from transformants had higher phosphate content compared with that of the control, on average 19%.

**Fig 5:**
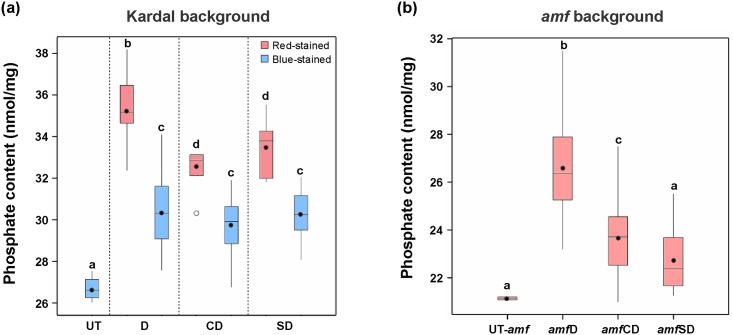
The phosphate content of starches from all transgenic series and control plants in both (a) Kardal and (b) *amf* backgrounds. D, CD and SD stand for transgenic series containing DSP protein, the full-length laforin protein and SBD-DSP fusion protein, respectively. *Amf* represents the amylose-free potato background. All the analyses were performed in duplicate on transformants with (engineered) laforin expression, which are 24, 25, 28, 32, 25 and 29 lines for D, CD, SD, *amf*D, *amf*CD and *amf*SD series, respectively. Significant difference between each transgenic group and control was analysed using one-way ANOVA. Different letters (a-d) indicate statistically significant differences at *p* < 0.05.

As shown in Fig 5a, in all series, the phosphate content significantly increased in starches from both red-stained and blue-stained groups compared with that from the control (ANOVA, *p* < 0.05). Within each series, the red-stained group had a higher phosphate content than the blue-stained group. To illustrate, the phosphate content of the red-stained group from D series was ~32% higher than that of the control, and ~9% and ~6% higher than the red-stained group from CD and SD series, respectively. In each of the three blue-stained groups, the starch phosphate content was ~11% higher than that of the control, while between the three series of transformants no significant differences were observed.

In the red-stained starches the increase in phosphate content appeared to be linked to the increase in amylopectin content rather than to the expressed laforin. However, blue-stained starches containing unaltered amylose content also showed a significant increase in phosphate content, which indicated that the (engineered) laforin plays a role to increase starch phosphate content. To further investigate this, the same constructs were transferred into amylose-free potato mutant (*amf*) and generated ~30 transformants of each transgenic series (*amf*D, *amf*CD and *amf*SD). Starch phosphate content was measured for each line. Results showed that the phosphate content in *amf*D and *amf*CD starches were 26% and 12% higher than that in control UT-*amf*, respectively (Fig 5b), while amfSD showed only a negligible increase. Overall, the increase in starch phosphate content in the *amf* background provides confidence that starch phosphate content is affected by the (engineered) laforin, and not only by GBSSI suppression.

### Granule size and gelatinization temperature are changed in modified starches

**Fig 6:**
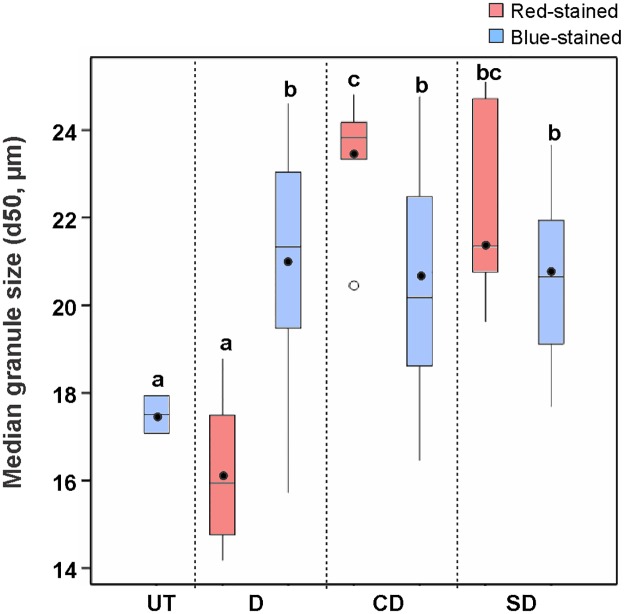
Boxplot presenting median granule size (d50) of starches from control UT and all transformants of each series. All the analyses were performed in duplicate on all transformants, which are 24, 25 and 28 lines for D, CD and SD series, respectively. Significant difference between each transgenic group and control was analysed using one-way ANOVA. Different letters (a-c) indicate statistically significant differences at *p* < 0.05.

The median granule size (d50) was determined for all starches by analysing granule size distribution. The transformants with CD and SD proteins had larger starch granule size compared to the control (Fig 6). In particular, starch granules of red-stained starches from CD series were significantly larger in size than that of blue-stained starches (ANOVA, *p* < 0.05). By contrast, starches from the red-stained group in D series did not show any significant difference in granule size. Further analyses showed that there was no significant correlation between the expression of the (engineered) laforin and granule morphology or granule size.

DSC analysis disclosed that red-stained starches had a significantly higher T_o_ and T_p_ (Table 1), suggesting that these starches start to gelatinize and swell to a maximum level at higher temperatures compared to the control. Moreover, the gelatinization enthalpy (Δ*H*) of these red-stained starches was consistently lower than that of the control. Unlike the red-stained starches, the gelatinization characteristics did not differ between the control and any of the blue-stained starches.

Starches, after debranching with isoamylase, were analysed with HPAEC (chain length ranged from DP6 to DP35). No significant changes in amylopectin fine structure were observed relative to the control (data not shown). Similarly, starch content was measured for the selected starches and no consistent changes were found compared to the control (Table 1).

### Starches in DSP transformants are different from those in CD and SD transformants

**Fig 7:**
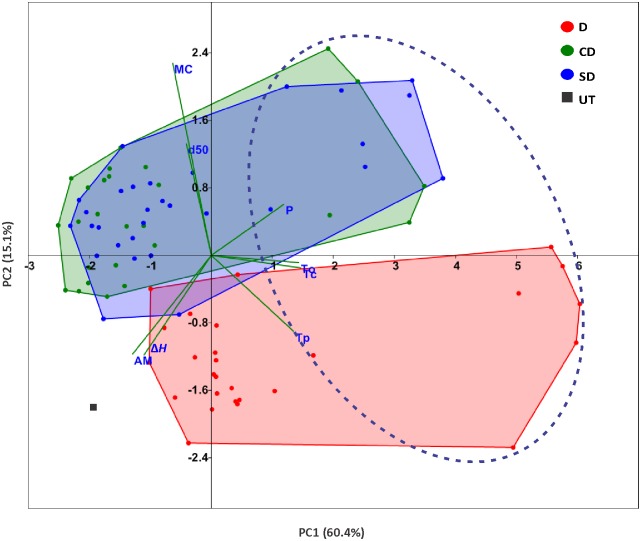
Principal components biplot displaying the classification of starches from three transgenic series based on the starch characteristics. Green vectors indicate the correlation between the different measured variables. P, phosphate content; T_o_, onset temperature of gelatinization; T_p_, peak temperature of gelatinization; T_c_, conclusion temperature of gelatinization; MC, starch moisture content; Δ*H*, gelatinization enthalpy; AM, amylose content; d50, granule median size.

To divulge more information resulting from combined effects of the different starch characteristics, a principal component analysis (PCA) was performed for all samples. As shown in Fig 7, the first component (PC1) explains ~60.4% of the variance and separated modified starches on their phosphate content (P), amylose content (AM) and gelatinization temperatures (T_o_, T_p_ and T_c_). The modified starches with relatively higher phosphate content, circled by the dotted line, had higher gelatinization temperatures, which is consistent with the visual inspection of the data in Table 1. The second component (PC2), which summarized ~15.1% of observed variation, was largely influenced by the variations in the starch moisture content (MC), gelatinization enthalpy (Δ*H*) and granule median size (d50). D series (red dots) was separated from CD and SD series (green and blue dots, respectively), while CD and SD modified starches did not separate from each other, suggesting that DSP functioned differently when alone than when appended to a glucan-binding domain (CD and SD). The analysis also showed that starches from CD and SD series had larger granules and higher MC than that from D series.

### Relationships between starch characteristics

**Fig 8:**
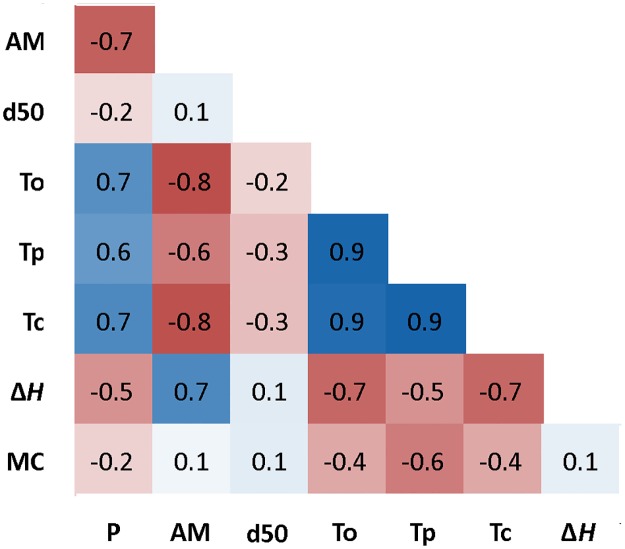
Heat-map displaying the extent and direction of correlations (*r*) between starch compositional characters and starch physico-chemical properties in transgenic lines. Correlations were statistically significant at *r* ≥ 0.22 and *r* ≤ -0.22. Blue colours show negative correlations and red colours show positive correlations. P, phosphate content; T_o_, onset temperature of gelatinization; T_p_, peak temperature of gelatinization; T_c_, conclusion temperature of gelatinization; MC, starch moisture content; Δ*H*, gelatinization enthalpy; AM, amylose content; d50, granule median size.

Correlation analyses were performed to dissect the underlying relationships between starch compositional characteristics and starch physico-chemical properties (Fig 8). Phosphate content was positively correlated (*r* = 0.6 or 0.7) with gelatinization temperatures (T_o_, T_p_ and T_c_), and negatively correlated with gelatinization enthalpy (Δ*H*, *r* = -0.5) and amylose content (AM, *r* = -0.7). In contrast, AM displayed remarkably opposite correlation patterns with P, exhibiting a negative association to T_o_, T_p_ and T_c_ (*r* = -0.8 or -0.6), and a positive correlation to Δ*H* (*r* = 0.7). Negative correlations between starch moisture content (MC) and gelatinization temperatures (*r* = -0.4 or -0.6) were observed. Furthermore, starch granule median size (d50) showed a negative correlation (*r* = -0.2 or -0.3) with T_o_, T_p_ and T_c_, but its associations with starch compositional characters (P, AM and MC) were not significant.

### Laforin affects expressions of starch dikinases and hydrolases

**Fig 9:**
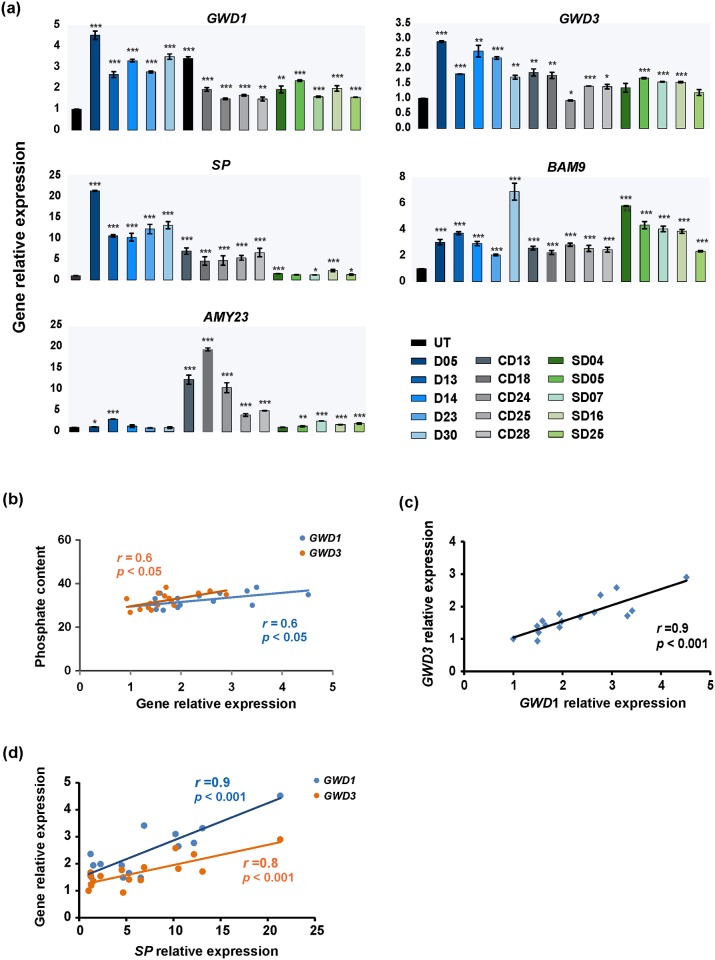
(a) The expression of the genes encoding key enzymes involved in starch metabolism and (b) correlation between starch phosphate content and gene expression and (c, d) between relative expressions of different genes. qRT-PCR was performed on control UT and 5 random-selected transgenic tubers from each series, containing transformants with 8 red-stained starches and 7 blue-stained starches. The expression level of following genes were measured: glucan, water dikinase (*GWD1*), phosphoglucan, water dikinase (*GWD3*), starch phosphorylase (*SP*), β-amylase 9 (*BAM9*) and α-amylase 23 (*AMY23*). The values are expressed as the mean ± S.D. from three independent measurements. Statistical significances between each starch sample and the control determined by using t-test (*, *p* < 0.1; **, *p* < 0.01; ***, *p* < 0.001).

To understand whether these differences in starch phosphate content were caused by direct or indirect action of laforin, expression levels of crucial genes responsible for starch phosphorylation was monitored by qRT-PCR on the same transformants previously selected to examine *GBSSI* expression. Interestingly, in general, the results displayed increased expression of starch dikinases, *GWD1* and *GWD3*, in all selected transformants compared to the control (Fig 9a). On the other hand, expression of orthologues of the *Arabidopsis SEX4*, *LSF1* and *LSF2* was not affected consistently in transformants (S2 Fig). Further analysis revealed that the expression level of both *GWD1* and *GWD3* was positively correlated with starch phosphate content (*r* = 0.6, *p* < 0.05, Fig 9b). In addition, a positive correlation (*r* = 0.9, *p* < 0.001) between the expression levels of both genes was found (Fig 9c), suggesting that they are co-regulated. Additionally, the expression level of these two genes was substantially higher in the D transformants than that in the CD, SD and the control, which is consistent with observed higher phosphate content.

To investigate whether the expression of genes involved in starch metabolic pathways was affected in the transgenic lines, four starch-degrading and seven starch-synthesizing genes were selected and their expression was assessed by qRT-PCR analysis. Generally, the expression level of starch-degrading genes was altered relative to the control, but various genes showed different patterns among transformants of the three different constructs (Fig 9a). The expression level of *SP* in the SD lines appears to be not much greater than in the controls, whereas the D and CD lines show much greater increases. Similarly, transformants from all series exhibited a significant increase in the expression of *BAM9* (t-test, *p* < 0.001). For the *AMY23* expression, an increase up to 20-fold (*p* < 0.05) was found in transfomants from CD series compared with the control, but the increase in transformants from D and SD series was smaller. For other starch-synthesizing genes no consistent differences between transformants and the control were detected for any of the series (S2 Fig). In addition, a positive correlation was established between *SP* expression and *GWD1* expression (*r* = 0.9), as well as *GWD3* expression (*r* = 0.8), indicating that these three genes are co-expressed (Fig 9d). Taken together, introduction of the (engineered) laforin leads to the up-regulation of starch-degrading genes, but does not affect the expression of genes involved in starch synthesis.

## Discussion

In this study, laforin, a human phosphatase gene, was engineered and introduced into potato plants to modulate phosphate content in starch granules, thus exploring the effect of phosphate content on starch properties and the mechanism of phosphoglucan metabolism in storage starch. When setting up the experiments, the idea was to generate starches with low amounts of phosphate content by expressing the (engineered) laforin gene in potato tubers. However, contrary to the expectation, the phosphate content increased substantially in starches from the transgenic lines compared with that of the control plants regardless of the construct and genetic background (Kardal and *amf*). This finding seems to conflict at first glance with the results of Worby, Gentry [32], who found that laforin dephosphorylates potato amylopectin *in vitro*. Clearly, the environment in potato amyloplasts is much more intricate than that in the *in vitro* system, and one important difference is the presence of other endogenous genes, which may be an obstacle to detecting the individual effect of laforin expression on phosphate content in potato. Therefore, we investigated whether the introduction of laforin affects the expression of other genes involved in starch phosphorylation pathway. Interestingly, the expression levels of starch phosphorylating genes, *GWD1* and *GWD3*, were significantly increased in the different transformants. Moreover, although modest, a positive correlation was found between starch phosphate content and the transcript levels of both *GWD1* and *GWD3* genes (Fig 9b). This result is consistent with the study of Smith, Fulton, Chia, Thorneycroft, Chapple, Dunstan [55], who found similar expression pattern for *GWD1* and *GWD3* genes in *Arabidopsis* leaves. Collectively, these results demonstrate that the expression of the (engineered) laforin in potato induces starch phosphorylation via GWD1 and GWD3 and thereby leads to an increase in phosphate content in starch granules, albeit the exact mechanism by which this would work remains to be elucidated and study on protein level is needed to further confirm the hypothesis. It has been demonstrated that starch phosphate groups are essential for well-functioning starch metabolism and plant performance (reviewed in [56]). It is therefore likely that a regulatory mechanism may exist to maintain certain proportional phosphate content in starch granules during starch synthesis. The phenomenon observed in this study could arise through a compensatory mechanism where laforin removes phosphate while *GWD1* and *GWD3* compensate by adding extra phosphate. Hence, the phosphate content obtained in modified starch might be the result of both (engineered) laforin activity and GWD1/GWD3 activity, and each of which is difficult to quantify. A similar compensatory regulation was also proposed by Mahlow, Hejazi, Kuhnert, Garz, Brust, Baumann [57], who detected higher GWD3 levels in the *Arabidopsis* GWD1-deficiency mutants.

One other clear effect of the transformation was that about 23% of modified starches displayed red-stained color and low amylose content, and further analysis revealed that the lower content of amylose was caused by GBSSI suppression (Fig 4). Moreover, these red-stained starches showed significantly higher phosphate content compared with that of blue-stained starches in each series (Fig 5, Table 1). A further increase in phosphate content is likely a consequence of the decreased amylose level, based on the fact that phosphate groups are covalently bound to amylopectin but not to amylose.

Notably, GBSSI suppression in transformants has been observed in earlier studies using similar constructs containing GBSSI promotor and transit peptide, however, this phenomenon occurred in a much lower ratio, about 3% [35, 58]. The authors suggested co-suppression of the *GBSSI* gene, which would also explain the results presented here, as the promoter and transit peptide used in the constructs were derived from the *GBSSI* gene. However, since in this study the occurrence rate of modified starches with low amylose content is dramatically higher than the above-mentioned studies, it suggests that the upregulation of *GBSSI* might also be a consequence of the presence of the (engineered) laforin.

Interestingly, an increase in the transcripts of starch-degrading genes in transformants was observed, but not in the starch-synthesizing genes (Fig 9a, S2 Fig). Furthermore, a positive correlation was found between the expression of the *SP* and that of phosphorylating genes, *GWD1* and *GWD3* (Fig 9d). These results re-enforce the view that the presence of phosphate content can stimulate starch degradation [3, 59, 60]. Although one would expect to observe a decrease in starch content, we haven’t been able to detect significant differences between transformants and the control.

Furthermore, CD and SD transformants did not show differences in starch characteristics, such as phosphate content, granule size, etc. These results indicate that replacing CBM20 with SBD in laforin did not affect the behaviour of the protein in potato. Intriguingly, D transformants had a higher starch phosphate content than CD and SD transformants. This might have resulted directly from the higher expression of *GWD1* and *GWD3* in D transformants (Fig 9a). Another possible reason could be the different phosphatase activity when expressing D alone or D associated with a starch-binding domain. Previous studies have shown that deleting CBM20 in laforin completely abrogated the phosphatase activity of laforin [61]. If this is the case, then differential turnover of starch phosphate content between D transformants and CD/SD transformants could be expected, leading ultimately to the differences in starch phosphate content.

Starch synthesis is a complex process, which is mediated by various enzymes. In this study, starch granules with altered morphology, irregular bumpy surface, and bigger size were observed in all series compared with the control. These changes are more likely to be caused by the effects of the (engineered) laforin on genes involved in starch biosynthesis/degradation rather than due to the direct activity of the (engineered) laforin enzyme. This might be the reason why no significant correlation has been found between laforin gene expression and starch granule morphology, size and properties. The CD and SD starch granules showed a larger size than the control, which is in accord with previous observations that the introduction of starch-binding proteins in potato could alter the granule morphology and granule size [33, 34, 36, 62].

Correlations between different starch compositional characters and starch properties have been identified. A strongly negative correlation (*r* = -0.7) was found between phosphate content and amylose content (in Kardal background), which is supported by previous studies [63–67]. These findings confirmed that phosphate content is dependent on amylose/amylopectin ratio. The correlation between phosphate content (P) and gelatinization characteristics might be an indirect effect caused by the decreased amylose content in modified starches, since we only found obvious changes on gelatinization characteristics in those starches with less amylose content (red-stained starches).

Overall, our results re-enforce the notion that alteration of starch components (amylose/ amylopectin ratio) in potato starch is an efficient way to modulate starch phosphate content in storage starch. Intriguingly, this study reveals that potato tuber starch, containing by nature already high level of starch phosphate, has obviously a strong compensatory mechanism to modulate starch phosphate content during starch biosynthesis. A better understanding of this compensatory mechanism may provide better insights on how to modify starch phosphate content in potato starch.

## Supporting Information

S1 Fig Schematic representation of pUC19/SBD2 vector. SBD, Linker, Amp and Ter stand for starch binding domain of cyclodextrin glycosyltransferase from *Bacillus circulans*, an artificial PT-linker, ampicillin resistant gene and terminator, respectively. To generate this construct, a sequence encoding the potato GBSSI promoter and part of GBSSI transit peptide (*Hind*III—*Hind*III) was amplified from the construct pBIN19/SBD2 [36] with primers 5’-CC**AAGCTT**AATACTAAAAAATGCAACAAAAT-3’ and 5’-CC**AAGCTT**GTTAACAGCCCTTAAACCAT-3’ and inserted into the corresponding sites of the pUC19 vector. The orientation of the *Hind*III—*Hind*III fragment was verified by sequencing. Subsequently, the sequence containing a SBD2 fragment and a part of GBSSI transit peptide (*Hpa*I—*Bam*HI) was amplified from pBIN19/SBD2 with primers 5’- C**GTTAAC**AAGCTTGATGGGCTCCAATCAAGAACT-3’ and 5’-CG**GGATCC**GCCAAAACAGCCAAGCTTATG-3’, followed by cloning into corresponding sites of pUC19.
(TIF)

S2 Fig The expression level of the genes encoding key enzymes involved in starch metabolism. Include: phosphoglucan phosphatase starch excess 4 (*SEX4*), like-SEX4 genes (*LSF1* and *LSF2*), starch-branching genes (*SBEI* and *SBEII*) and isoamylase genes (*ISA1*, *ISA2* and *ISA3*), soluble starch synthase genes (*SSSII* and *SSSIII*) and β-amylase 1 (*BAM1*). The qRT-PCR was performed on control tubers (UT) and five random-selected transgenic tubers from each series, containing transformants with eight red-stained starches and seven blue-stained starches. The values are expressed as the mean ± S.D. from three independent measurements. No consistent changes in the expression level of these genes were observed relative to the control.
(TIF)

S1 Table The qRT-PCR primer sequences of genes of interest and one reference gene. (PDF)

S2 Table The list of abbreviations. (PDF)
