# Supplementary figures and images for: Engineering Potato Starch with a Higher Phosphate Content
Source: PLoS One. 2017 Jan 5;12(1):e0169610. doi: 10.1371/journal.pone.0169610 (PMC5215930; doi:10.1371/journal.pone.0169610)

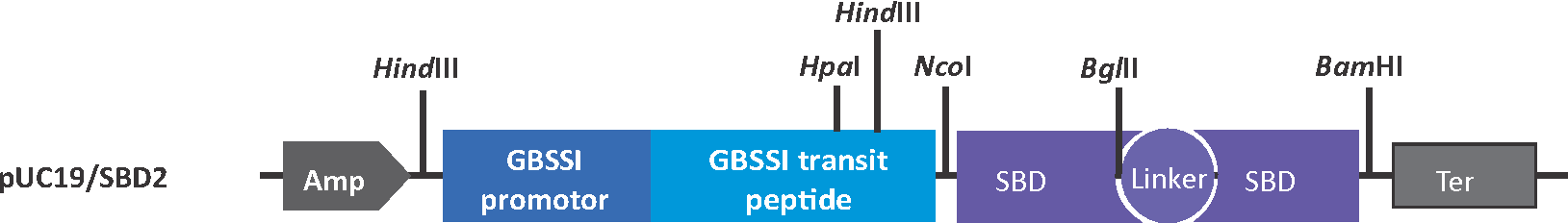

Supplement: S1 Fig — SBD, Linker, Amp and Ter stand for starch binding domain of cyclodextrin glycosyltransferase from Bacillus circulans, an artificial PT-linker, ampicillin resistant gene and terminator, respectively. To generate this construct, a sequence encoding the potato GBSSI promoter and part of GBSSI transit peptide (HindIII—HindIII) was amplified from the construct pBIN19/SBD2 [36] with primers 5’-CCAAGCTTAATACTAAAAAATGCAACAAAAT-3’ and 5’-CCAAGCTTGTTAACAGCCCTTAAACCAT-3’ and inserted into the corresponding sites of the pUC19 vector. The orientation of the HindIII—HindIII fragment was verified by sequencing. Subsequently, the sequence containing a SBD2 fragment and a part of GBSSI transit peptide (HpaI—BamHI) was amplified from pBIN19/SBD2 with primers 5’- CGTTAACAAGCTTGATGGGCTCCAATCAAGAACT-3’ and 5’-CGGGATCCGCCAAAACAGCCAAGCTTATG-3’, followed by cloning into corresponding sites of pUC19. (TIF) [file pone.0169610.s001.tif]

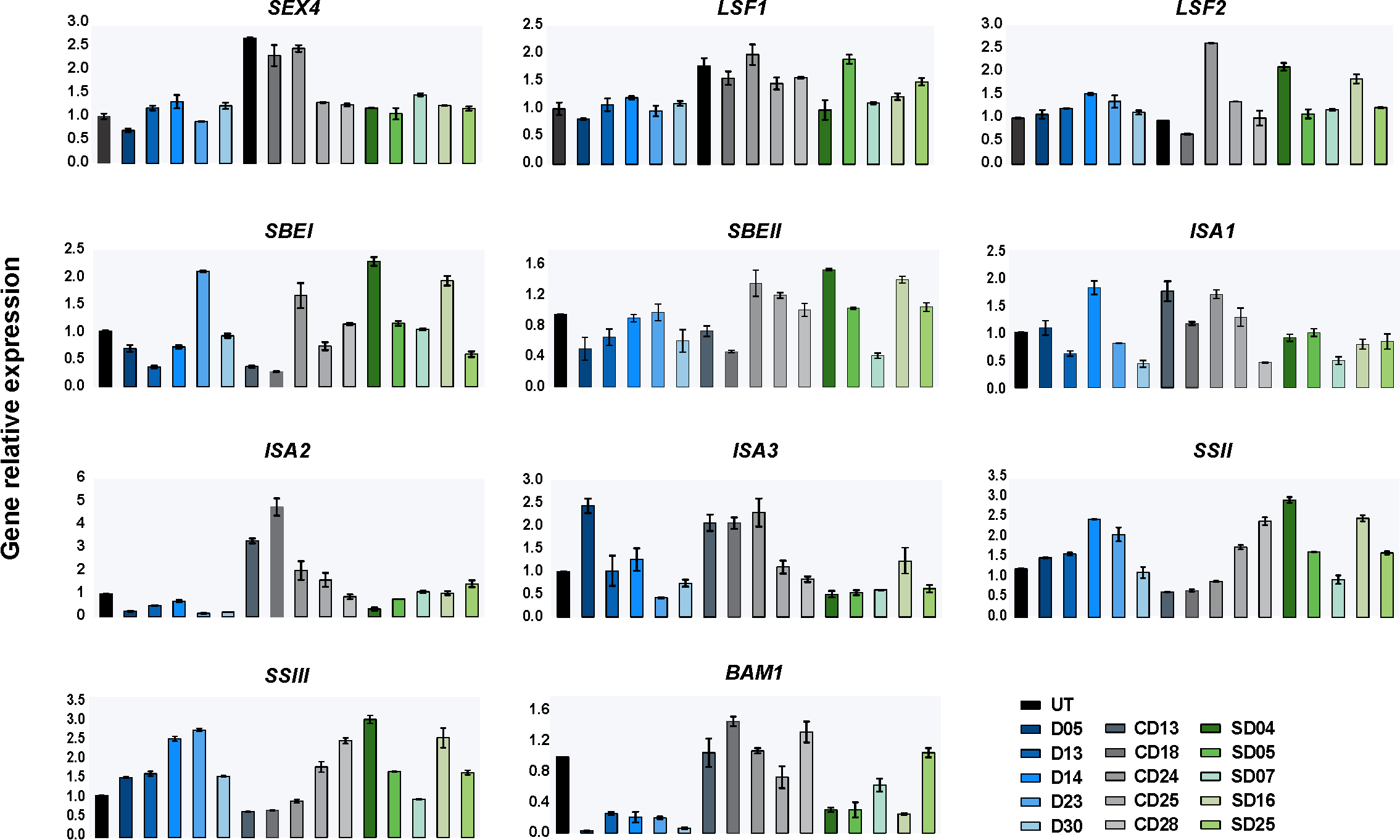

Supplement: S2 Fig — Include: phosphoglucan phosphatase starch excess 4 (SEX4), like-SEX4 genes (LSF1 and LSF2), starch-branching genes (SBEI and SBEII) and isoamylase genes (ISA1, ISA2 and ISA3), soluble starch synthase genes (SSSII and SSSIII) and β-amylase 1 (BAM1). The qRT-PCR was performed on control tubers (UT) and five random-selected transgenic tubers from each series, containing transformants with eight red-stained starches and seven blue-stained starches. The values are expressed as the mean ± S.D. from three independent measurements. No consistent changes in the expression level of these genes were observed relative to the control. (TIF) [file pone.0169610.s002.tif]
