# Supplementary material for: Engineering Potato Starch with a Higher Phosphate Content
Source: PLoS One. 2017 Jan 5;12(1):e0169610. doi: 10.1371/journal.pone.0169610 (PMC5215930; doi:10.1371/journal.pone.0169610)
Supplement: S1 Table — (PDF) [file pone.0169610.s003.pdf]

S1 Table. **The qRT-PCR primer sequences of genes of interest and one reference gene.**

Gene	Gene ID	Forward primer 5'-3'	Reverse primer 5'-3'	Reference
**Laforin**	AF084535.2	attcctcaggctgtaaccgctac	gctcatatctggtgttggcatcc	
GBSSI	X58453	gagcttctggcagtgaaccc	ggcaagtggagcgatttcttc	Kok-Jacon *et al.* (2005)
GWD1	AY027522.1	gggtggtcttttgcgtgatt	tgtagcccatgcagtttgca	Chen *et al.* (2012)
GWD3	GU045560	caatagctatgcgtcggaagtg	gctttgcattcctcgggcttc	Orzechowski *et al.* (2013)
SEX4	PGSC0003DMG400015246*	tcaaaagtgccacagcagacatcc	aactccctctggagagtccacaaacc	
LSF1	PGSC0003DMG400030092*	ccacaaaggtggtccaagatttgag	ttgcgtccttcttttgctgttgaatg	
LSF2	PGSC0003DMG400029073*	tcaattgcttatatgttctggttctgtg	atcagtttcctctcccaatctgctac	
AMY23	M79328	ggcatacacagccgttcatct	atccgtccccaatcttcacg	Ferreira (2011)
BAM1	PGSC0003DMT400003933*	tgagatgcgtgaccatgagc	caagtggaacttgcgcttcc	Zhang *et al.* (2013)
BAM9	PGSC0003DMT400027659*	cttgatggaaagactccgg	agaaacgcccgtgattgtg	Zhang *et al.* (2013)
SP	X52385	caggaaccagatgctgctctt	catagccccatgctgggtagt	Zhang *et al.* (2013)
ISA1	102577466	ggcaaatggagaggacaaca	atgggaacaccttgggaaac	Bustos *et al.* (2004)
ISA2	102577931	ttatccttccgccacctc	cttcaactggagttcccttct	Bustos *et al.* (2004)
ISA3	102577824	gacgcttgcccttcattc	ctcctgtgcggttcttctgt	Ferreira (2011)
SBEI	102596498	ccgagccccacgaatctat	ggctcagagctgctcatgc	Safford *et al.* (1998)
SBEII	102590711	actcgcaactgatgtggatg	ggcattccgctaacatcttc	Jobling *et al.* (1999)
SSSII	102583115	ggcaagcctcaatgtaaagc	acacccttttgtgggtcaag	Edwards *et al.* (1995)
SSSIII	102577674	cacaggaggtgtctggaaacc	tggaacttgtgaaggtgaggc	Abel *et al.* (1996)
EF1α	AB061263	attggaaacggatatgctcca	tccttacctgaacgcctgtca	Nicot *et al.* (2005)

* Sequence ID from the Potato Genome Sequence Consortium database.
