# Supplementary material for: Engineering Potato Starch with a Higher Phosphate Content
Source: PLoS One. 2017 Jan 5;12(1):e0169610. doi: 10.1371/journal.pone.0169610 (PMC5215930; doi:10.1371/journal.pone.0169610)
Supplement: S2 Table — (PDF) [file pone.0169610.s004.pdf]

S2 Table. The list of abbreviation.

DSP	dual-specificity phosphatase
CBM20	carbohydrate-binding module 20
SBD	Starch-binding domain
D	Transgenic lines containing DSP
CD	Transgenic lines containing full-length laforin protein
SD	Transgenic lines containing DSP with an SBD
amf	Amylose-free potato mutant
P	Phosphate content
AM	Amylose content
C_{starch}	Starch content
d50	Median granule size
To, Tp and Tc	Gelatinization temperature
ΔH	Gelatinization enthalpy
MC	Starch moisture content
GBSSI	Granule-bound starch synthase I gene
GWD1	Glucan, water dikinase gene
GWD3	Phosphoglucan, water dikinase gene
SP	Starch phosphorylase gene
BAM1 and *BAM9*	β-amylase genes
AMY23	α-amylase 23 gene
SEX4, *LSF1* and *LSF2*	Starch phosphatase gene
SSII and *SSIII*	Starch synthase genes
SBEI and *SBEII*	Starch branching genes
ISA1, *ISA2* and *ISA3*	Isoamylase genes
EF1α	Elongation factor gene
